# Draft Genome Sequences of 11 Bacterial Strains Isolated from Commercial Corn-Based Poultry Feed

**DOI:** 10.1128/MRA.00170-20

**Published:** 2020-04-16

**Authors:** Elena Olson, Andrew Micciche, Joseph L. Sevigny, Steven C. Ricke, Anuradha Ghosh

**Affiliations:** a Department of Biology, Pittsburg State University, Pittsburg, Kansas, USA; b Center for Food Safety, Department of Food Science, University of Arkansas, Fayetteville, Arkansas, USA; c Hubbard Center for Genome Studies, University of New Hampshire, Durham, New Hampshire, USA; Indiana University, Bloomington

## Abstract

Here, we report 11 bacterial strains isolated from commercial corn-based poultry feed to determine their potential as hygienic indicator microorganisms through a comparison of genome sizes and distribution patterns of unique genes. These isolates belonged to the genera *Klebsiella*, *Kosakonia*, *Pantoea*, *Stenotrophomonas*, and *Enterococcus*.

## ANNOUNCEMENT

The microbial composition of poultry feed could be a critical factor for the development and growth of broilers and the establishment of their gastrointestinal tract (GIT) microbiome, particularly for those given feeds treated with antimicrobials, such as formaldehyde (1). Poultry feed harbors a wide array of microorganisms, including some potential pathogens (2). However, little is known about the taxonomy of nonpathogenic bacteria associated with commercial feeds. Since the presence of pathogens is relatively infrequent, it is important to gain a better understanding of the distribution and prevalence of nonpathogens (1, 2). Hygienic indicator organisms, represented by total aerobic colony count and coliform count, as well as *Enterobacteriaceae*, function in a way similar to that of particular foodborne pathogens and thus offer a permanent method for assessing and predicting the efficiency of sanitization agents against consequent pathogens that are difficult to detect (2).

Based on previous findings, next-generation sequencing based on 16S rRNA gene amplification has been proposed for use in characterizing microbial populations in poultry feeds (2, 3). Therefore, application of whole-genome sequencing (WGS) has been widely accepted for predicting possible microbial threat or preventing premature product spoilage under the food safety purview (4). WGS analysis performed on nonpathogenic and potentially pathogenic poultry feed isolates in this study will facilitate understanding of the distribution of candidate genes encoding proteins/enzymes related to virulence, toxins, stress, antimicrobial resistance, porins, monooxygenases, oxidoreductases, dioxygenases, and catabolism of heavy metals (lead, arsenic), among others, and will eventually determine the most suitable hygienic indicator bacteria in the poultry processing pipeline.

Bacterial isolates from corn-based chicken feed were recovered on aerobic plate count (APC) agar (5). Initially, 10 g of feed was shaken in 100 ml tryptic soy broth (TSB) (BD Difco, Franklin Lakes, NJ) for 2 minutes; the mixtures were serially diluted and plated onto APC agar. Isolates were grown overnight at 37°C in an incubator. Unique colonies were isolated and purified repeatedly (3 times). Finally, 11 morphologically different colonies were selected for WGS analysis. Genomic DNA was extracted from pure cultures grown overnight in TSB at 37°C in an incubator using a DNase blood and tissue kit for bacteria (Sigma-Aldrich Corporation, Natick, MA) following the manufacturer’s protocol (http://www.bea.ki.se/documents/EN-DNeasy%20handbook.pdf). A Nanodrop lite (Thermo Fisher Scientific, Waltham, MA) analysis was performed on each isolated DNA sample for quantification purposes and to ensure sample purity (5).

WGS was performed at the Hubbard Center for Genome Studies (University of New Hampshire, Durham, NH). A paired-end library was constructed using a Nextera DNA library preparation kit (Illumina, San Diego, CA) and sequenced with an Illumina HiSeq 2500 instrument to produce 250-bp paired-end reads. The total number of reads for each of the 11 strains is listed in Table 1. Fastq files were trimmed for Nextera adapters and low-quality bases using Trimmomatic version 0.32 (6). For read trimming, trailing and leading bases were removed if the quality score was below 3. In addition, the reads were scanned using a 4-base sliding window and trimmed if the average quality dropped below 15. Trimmed sequencing reads were then assembled using the SPAdes pipeline version 3.5 (7) with default settings. QUAST version 4.6.0 (8) was used to assess the contiguity of the assemblies, and coverage statistics were calculated by mapping fastq reads to the assembled contigs with BWA-MEM (default settings) (9). The assembled genomes were annotated via the NCBI Prokaryotic Genome Annotation Pipeline (PGAP) (10). The taxonomic identity of each isolate was further confirmed by performing a BLASTn (11) search on 16S rRNA (∼1,500 bp) and translation initiation factor 2 (IF-2; ∼2,750 bp) gene sequences against the NCBI nucleotide database. For all isolates, the species identities ranged between 99% and 100%. The assembly metrics and annotated features are given in Table 1.

**TABLE 1:** Accession numbers, assembly metrics, and annotated features of the sequenced strains isolated from commercial corn-based chicken feed

Bacterial species	Strain	GenBank accession no.	SRA accession no.^*a*^	Avg coverage (×)	No. of contigs	Total no. of reads	Genome assembly size (bp)	*N*_50_ (bp)	G+C content (%)	No. of coding genes	No. of rRNAs	No. of tRNAs	No. of ncRNAs^*b*^
*Enterococcus* sp.	PF-2	VFLR00000000	SRS4994585	425	29	6,481,210	3,667,502	436,646	53.30	3,350	6	51	4
*Enterococcus* sp.	PF-3	VFLT00000000	SRS4994583	1,003	29	15,261,978	3,667,758	516,576	43.74	3,351	8	51	4
Klebsiella variicola	PF-5	VFLW00000000	SRS4994578	348	30	8,094,380	5,548,017	446,212	57.35	5,222	15	78	9
Klebsiella variicola	PF-1	VFLS00000000	SRS4994582	405	32	10,377,412	5,548,808	409,857	56.66	5,222	15	78	9
Kosakonia cowanii	PF-6	VFLX00000000	SRS4994579	340	24	7,202,962	4,806,877	532,407	56.11	4,381	9	74	11
Kosakonia cowanii	PF-9	VFMA00000000	SRS4994586	409	25	8,787,018	4,807,035	532,407	56.06	4,381	9	76	11
Kosakonia cowanii	PF-104	VFLU00000000	SRS4994580	435	25	9,139,632	4,807,035	532,407	54.78	4,384	9	76	11
Pantoea vagans	PF-103	VFLQ00000000	SRS4994584	510	19	9,486,610	4,573,523	527,489	53.64	4,180	15	70	6
Pantoea vagans	PF-7	VFLY00000000	SRS4994576	420	19	8,755,260	4,696,349	584,032	53.22	4,253	16	69	13
Stenotrophomonas maltophilia	PF-8	VFLZ00000000	SRS4994577	530	28	11,071,680	4,520,531	405,440	65.89	4,053	7	64	4
Stenotrophomonas maltophilia	PF-4	VFLV00000000	SRS4994581	485	26	9,405,034	4,377,836	351,312	66.15	3,878	7	64	4

a SRA, Sequence Read Archive.

b ncRNAs, noncoding RNAs.

### Data availability.

Draft genome sequences and raw sequencing reads have been deposited at DDBJ/ENA/GenBank under the BioProject accession number PRJNA543860, and the described accession numbers in this publication are listed in Table 1.
